# Operationalizing Accountability for Integrated Care: A Qualitative Study of an Ontario Health Team

**DOI:** 10.1177/08404704251382221

**Published:** 2025-10-08

**Authors:** Nusrat Farhana, Kian Rego, Jenna M. Evans

**Affiliations:** 1 Dalla Lana School of Public Health, 206712 University of Toronto, Toronto, Ontario, Canada; 2 DeGroote School of Business, 120456 McMaster University, Hamilton, Ontario, Canada

## Abstract

Shared accountability is widely emphasized in integrated care theory and policy but remains underspecified in practice. This study examined how shared accountability was operationalized and experienced within an Ontario Health Team (OHT) using data from 23 semi-structured interviews with OHT stakeholders. Six interrelated factors that shape shared accountability were identified: perceived organizational identity, clarity of leadership roles and consequences for non-compliance, clarity of partner organizations’ roles and consequences for non-compliance, management of goals and interests, trust and psychological safety, and power dynamics. Together, these factors highlight that shared accountability is not merely a matter of assigning roles or measuring outcomes; rather, it is a complex, relational process. These findings offer practical guidance for strengthening shared accountability in integrated care networks.

## Introduction

Integrated care relies on coordinated efforts across sectors, organizations, and providers to deliver more seamless and patient-centred services.^1^ Achieving this coordination requires robust governance mechanisms that align goals, distribute responsibility, and support joint decision-making and action.^2,3^ Among these mechanisms, shared accountability is frequently emphasized in policy and practice as a cornerstone of integrated care.^4-9^ Shared accountability involves multiple actors assuming joint responsibility for performance goals and outcomes.^3,10^ Establishing shared accountability requires new structures that may be enacted formally and hierarchically—through contracts, mergers, or performance frameworks—or informally and horizontally, through partnerships and mutual commitments.^6,10^

Despite its prominence in policy and theory, shared accountability is rarely examined empirically in the integrated care literature.^9^ It is commonly described in broad terms, such as joint responsibility for care quality and outcomes, but with limited exploration of the specific processes, structures, and lived experiences that give the concept meaning in practice.^9^ This article helps address that gap by exploring how shared accountability was enacted and experienced in an integrated care initiative. Using a case of an Ontario Health Team (OHT), we asked: How is shared accountability operationalized in integrated care initiatives, and what factors shape how it is experienced by those involved?

## Methods

### Policy Setting

OHTs were launched in 2019 as a new model for integrated care in Ontario.^11^ Each OHT is composed of groups of providers such as hospitals, primary care providers, home and community care services, and mental health and addictions agencies, who voluntarily collaborate as partners with support from a small backbone team (e.g., an executive lead, project manager(s), and administrative staff).^11^ OHTs are accountable to Ontario Health, the provincial body that oversees the health system. OHTs receive $750,000 annually from Ontario Health. In addition to meeting OHT-specific community needs, each OHT is expected to contribute to health system goals, operationalized through a collaborative Quality Improvement Plan (cQIP) submission to Ontario Health, which requires the establishment of justifiable performance targets and continued progress towards meeting the targets.^12^

Although OHTs are collectively responsible for integrating care for their attributed populations, they remain separate from their partner organizations. OHTs are not legal entities and therefore cannot hold funds or employ staff directly; these functions are usually fulfilled by a partner organization. Partner organizations retain their respective funding and accountability relationships and do not receive OHT-specific compensation for their participation.

Ontario Health offered minimal direction on how integration should occur, including how governance and accountability structures should be designed. Each OHT was expected to develop governance arrangements that are “fit for purpose”^13^ and to create a Collaborative Decision-Making Arrangement (CDMA)—a formal non-legally binding document outlining expectations for financial management, resource allocation, performance monitoring, information sharing, and conflict resolution.^14^ However, while CDMAs must be submitted for approval, their implementation is largely unmonitored, and the extent to which they are actively used to shape decision-making and performance accountability varies across OHTs. Broader accountability structures were also underdefined and left to the discretion of each OHT.

### Study Setting

This study was based on one OHT where one of the authors worked as an embedded researcher. The OHT serves an attributed population size of over 100,000 with a mixture of urban and rural geography and has a comprehensive range of partner organizations representing primary, acute, long-term, palliative, mental health and community/social care services as well as public health and local municipalities. The OHT established a Collaboration Council composed of representatives from partner organizations and is co-chaired by two partner organization leaders to provide strategic leadership and collective governance of the OHT.

At the time of the interviews, the OHT backbone team was composed of four staff in project management and administrative support, and one Executive who led the team. Since the OHT is not a legal entity, one partner organization signed the Transfer Payment Agreement (TPA) on behalf of the backbone team and provided back-office support for a fee. The entire backbone team became official employees of the TPA holder, although the non-executive staff reported to the OHT backbone’s executive leader. The backbone team’s Executive leader was accountable to the Collaboration Council.

### Data Collection and Analysis

Between July-November 2023, 23 semi-structured informant interviews of 45-60 minutes in length were conducted with the OHT’s stakeholders as part of a quality improvement initiative. The goal was to better understand team members’ perceptions of OHT functioning and use these insights to guide improvements in day-to-day operations. The participants included perspectives of patient and family/caregivers (n = 2), physicians (n = 1), OHT backbone team members (n = 1), and partner organization leaders (n = 19). Of the 19 partner organization leaders, all but one were in senior roles, with the majority being Executive Directors, Vice-Presidents, or CEOs. The interviews were conducted by the embedded researcher—a postdoctoral fellow with training and experience in interviewing and field note documentation. Interviews focused on participant perceptions of OHT functioning, including care integration, performance measurement, information sharing, and partner relations. In-depth notes were taken during the interviews that included descriptions of participant views and experiences as well as verbatim quotations. Participants were contacted for follow-up data clarifications, as needed. Since this project was a quality improvement exercise, in accordance with the *Tri-Council Policy Statement: Ethical Conduct for Research Involving Humans* (2022),^15^ ethics approval was not required. Nonetheless, verbal informed consent was obtained from all participants, and procedures for confidentiality and data security were upheld at every stage of the project.

Interview notes were organized and coded in Microsoft Excel during the initial round of analysis to identify a broad set of themes, which were presented to the OHT to support quality improvement. Accountability was identified as a recurring cross-cutting issue, after which the notes were re-examined and re-coded in Microsoft Excel with a more focused lens on participants’ perceptions of accountability, generating six themes. Coding and theme development were conducted by two researchers, who met to compare codes and resolve discrepancies.

## Results

Six interrelated factors were identified that influence how shared accountability is operationalized in the OHT. Each factor is described further below and outlined in Table 1 with representative quotes and key self-assessment questions for OHTs.

**Table 1.** Key Factors Shaping Shared Accountability in OHTs: Guiding Questions and Examples from the Data

Factor	Guiding questions	Illustrative examples
Perceived organizational identity	• To what extent do participants identify with the OHT relative to their organization?	“We need to think of ourselves as an integrated system rather than a gathering of organizations” [P22]
	• How do participants perceive their identity when they are members of more than one OHT?	“[The] OHT is responsible for doing certain activities that the OHT needs to demonstrate that they have been able to accomplish with or without the support of partners” [P18]
Clarity of leaders’ roles and consequences for non-compliance	• What are OHT backbone leaders responsible for vs. partner organization leaders?	“How do you hold each individual accountable for their participation and engagement? We have the CDMA, we have the Relationship Charter, which is all a good place to start. Who’s to do that when we’re all colleagues? Like there’s one thing for me to hold my managers and my staff accountable because I have authority over them” [P12]
	• What consequences are in place, if any, for neglecting one’s role?	“If you continually underperform in supporting the OHT, you can be removed from the OHT. But then once you do that you are now putting a hole in your OHT. Now you are missing a partner” [P22]
Clarity of partner organizations’ roles and consequences for non-compliance	• To what extent can participants “see” how the partner organizations fit together and their relative and combined contributions, for example, via shared medical records, care plans, or performance reports?	“…It [is] important for me to understand across the OHT what are the other organizations doing to support that same population to understand the overlay; what are the contributions we are all making to drive the success of [that] indicator” [P4]
	• What consequences are in place, if any, for a lack of contribution?	“If each organization has to participate in 20 meetings a year, that’s fine, but if each organization has to contribute 5% of their budget that’s where you are going to run into issues because not everybody has the same [resources]” [P11]
		“Each organization that is coming to the table is governed by their own boards and the OHT is in the middle without really any governance structure…Agencies are coming and the work is being done off the side of their plates” [P4]
Management of goals and interests	• To what extent are goals/outcomes and associated measures shared across OHT partners?	“Each organization is going to have their own metrics that are reportable but […] [for OHT indicators] we need to identify what are the joint shared responsibilities” [P22]
	• What is in the best interests of the OHT vs. individual organizations and what incentives shape how participants prioritize these interests?	“There has to be a willingness [among partner organizations] to come to the table and see these cQIP indicators as just as important as their own individual agency indicators” [P14]
Extent of trust and psychological safety	• To what extent do participants feel safe sharing problems and ideas and asking for help from partner organizations or OHT backbone leaders?	“We all have a part in reviewing and helping each other…That is where trust comes in. We have all got to be able to give each other feedback” [P4]
	• Are both accountability and psychological safety optimized?	“It’s about creating a safe environment that leaders can feel that it’s okay to be criticized about your organization and that people become comfortable about approaching other organizations [to say] maybe this needs to be looked at differently to increase our relationship that will benefit the client” [P9]
Power dynamics	• What levers do OHT backbone leaders have, beyond moral suasion, to enforce change across partner organizations?	“There needs to be a lever of accountability to the OHT that supersedes the partner organizations' existing accountabilities…Because [partner organizations'] CEOs have a fiduciary responsibility to their own organizations” [P2]
	• How do differences in partner organizations’ size, resources, location, reputation, influence relations and decisions?	“We need to break it down in a way that allows budgets to shift or people to shift without the accountability [to respective funders/ministries] being a barrier” [P2]
		“No matter what you do, the hospital is still the centre of the healthcare world, right? And it gets the most funding and has the largest staff...A lot of what [social services] are measured on is in relation to hospital [utilization]” [P12]

### Perceived Organizational Identity

The degree to which participants identify with the OHT, relative to their own organization, shapes how they experience shared accountability. While some conceptualized the OHT as one entity composed of the backbone team and partner organizations, others saw this as an ideal rather than a reality. Each partner organization brings its own mission, norms, and ways of working, which must be negotiated to create a shared sense of purpose. Misaligned identities—driven by differences in mandates, funding models, ministry accountabilities, or organizational cultures—can make shared accountability difficult to achieve or sustain. This challenge is further complicated when organizations participate in multiple OHTs, making it harder to define their role, commitments, and alignment within each one.

### Clarity of Leaders’ Roles and Consequences for Non-Compliance

Clearly defining leadership roles and establishing consequences for non-compliance are critical for operationalizing shared accountability. In the OHT, backbone leaders are generally expected to report to government, implement directives, coordinate partner engagement, manage budgets, and secure funding, among other tasks. In contrast, partner organization leaders oversee their own institutions while contributing to the Collaboration Council, joining working groups, offering in-kind support, and co-developing integrated care pathways. When care pathways exist, they are expected to align their organization’s practices accordingly—a task that can involve significant organizational change.

However, these roles are often dynamic and may not be codified in the CDMA, and consequences for not fulfilling them are unclear. The Collaboration Council can hold the backbone leader accountable, including through dismissal. However, the backbone leader lacks reciprocal authority: they cannot remove partner leaders without risking the loss of a vital partner and weakening the collaborative. This imbalance makes it difficult to enforce shared accountability consistently across all parties.

### Clarity of Partner Organizations’ Roles and Consequences for Non-Compliance

Shared accountability depends on a clear understanding of what each partner organization is expected to contribute and how those contributions fit together to achieve integrated care. Participants emphasized the importance of seeing the collective impact of organizational contributions—such as through shared records, care plans, or performance data—but noted that this systems-level view remains limited. Instead, many organizations continue to prioritize internal mandates over shared goals.

While partner contributions are essential, they are often driven by goodwill rather than formal accountability. OHTs largely function as coalitions of the willing, with few mechanisms to ensure that organizations meet their commitments. Resource limitations, particularly for smaller organizations, further constrain their capacity to contribute time, staff, or data. Administrative and legislative barriers, such as restrictions on information sharing and rigid funding rules, also hinder collaboration and make it difficult to monitor or enforce accountability across partners.

### Management of Goals and Interests

Achieving shared accountability requires clarity on both the individual goals of partner organizations and the collective goals of the OHT. While each organization operates under its own mandate, certain outcomes, such as reduced Emergency Department (ED) visits, management of mental health and addictions in the community, and improved patient experience, were widely viewed as shared OHT objectives. These broader outcomes align with the OHT’s emphasis on community-level health and continuity of care, rather than organization-specific service metrics like referral counts, which were largely criticized by participants as inadequate measures for integrated care.

Despite this alignment in principle and the existence of well-defined performance indicators at the individual partner organization level, which goals are truly shared, and how to operationalize and measure each partner organization’s contribution to the shared goals remains largely underdeveloped. This makes it difficult for partners to see how their efforts contribute to collective outcomes and to identify the missing link in the care chain when outcomes are not achieved. Participants highlighted the need for a shared performance measurement system that captures experience and outcomes data on patients’ health and social care journeys. However, limitations in data access and infrastructure hinder progress on this front.

Participants also emphasized the importance of distinguishing between what benefits individual organizations and what advances the interests of the OHT as a whole. Without clear incentives or mandates, organizations often prioritize internal goals over collective ones, especially when integration efforts risk reducing their visibility, funding, or autonomy. Since formal accountability mechanisms are weak, moral suasion remains the primary means of alignment, leaving the system vulnerable to disengagement when interests conflict. At the same time, incentives, such as financial pressures or reputational benefits, can motivate collaboration. For shared accountability to be sustained, incentives must reinforce the value of collective goals alongside organizational priorities.

### Extent of Trust and Psychological Safety

Shared accountability depends not only on formal structures, but also on a culture where leaders feel safe acknowledging challenges, offering feedback, and asking for help—both from the backbone team and from (and among) partner organizations. However, some noted that trust can be fragile, and giving or receiving critical feedback can still feel uncomfortable.

Psychological safety allows leaders to surface problems without fear of blame and to collaboratively explore solutions that benefit the collective. This is especially important when addressing performance issues or navigating inter-organizational tensions. Fostering trust and psychological safety strengthens relationships, supports continuous learning, and reinforces a sense of shared ownership for OHT outcomes.

### Power Dynamics

Power dynamics refer to how influence is distributed and exercised, shaping participation, decision-making, and accountability. Power shapes who drives decisions, who can be held accountable, whose priorities take precedence, and whose contributions are visible and valued. Participants voiced that these dynamics are influenced by differences in authority, resources, sectoral dominance, and the OHT’s governance structure.

At the individual level, uneven professional risks were noted: while the OHT backbone leader can be removed from their role, partner organization leaders may remain employed within their own institutions regardless of OHT engagement. Without formal authority or vertical accountability, the backbone leader must rely on peer influence and moral suasion. The absence of formal governance structures, such as an OHT board, further limits enforcement capacity. Conversely, if the Collaboration Council is weak or not invested in attaining OHT goals, the absence of an OHT board may also dampen holding the OHT backbone executive leader to account for deliverables.

At the organizational level, differences in size, infrastructure, and funding create imbalances. As participants noted, larger organizations, particularly hospitals, often dominate due to greater resources and control over performance data, reinforcing hospital-centric metrics. Smaller organizations may struggle to contribute fully, increasing their dependency on both the OHT and larger partners. Simultaneously, the backbone team—lacking incorporation or independent infrastructure—may depend on well-resourced partners for operational support. This interdependence can invert expected accountability relationships and compound existing imbalances.

## Discussion

This study identified six interrelated factors that influenced how shared accountability was operationalized and experienced in an OHT. Together, these six factors reveal that shared accountability in integrated care initiatives is not simply a matter of assigning responsibilities or tracking outcomes; it is a complex and relational process. Perceived organizational identity determines how strongly participants align with the OHT’s collective purpose, while clarity around both leader and partner roles defines who is responsible for what, and what happens when those responsibilities are not fulfilled. Goal management links these roles to tangible outcomes and highlights the need for aligned incentives that balance organizational and collective interests. Trust and psychological safety form the relational foundation that allows partners to address performance challenges openly and constructively. Finally, power dynamics, both formal and informal, cut across all other factors, influencing how decisions are made, whose voices are amplified, and whether shared accountability is truly possible.

These findings echo and extend existing literature. Prior work suggests that strong pre-existing professional and organizational identities can hinder inter-organizational collaboration^16^ and that cultivating an “inter-group relational identity” can support a sense of belonging without erasing professional and organizational distinctiveness,^17^ helping to reinforce shared accountability. While the literature notes the importance of role clarity to achieving shared accountability,^18^ particularly in integrated care efforts,^19^ there is limited guidance on how to hold individuals and organizations to account for disengagement or non-compliance—a prominent theme in our results. Many studies of integrated care research emphasize the need for team- or network-level performance indicators that capture joint contributions and shared client-centred outcomes.^20,21^ Trust and psychological safety have also long been recognized as foundational conditions for collaboration and accountability.^22,23^ However, structural inequities, such as resource disparities and sectoral dominance, can erode trust,^20,24^ making it harder to rely on informal accountability mechanisms like moral suasion. Finally, while integrated care initiatives often aim for distributed leadership^25^ our findings reinforce the importance of addressing both relational and structural power imbalances to create more equitable conditions for shared accountability.^19,26,27^

## Limitations

This project was conducted within a single OHT, which may limit the transferability of the findings to other OHT and integrated care settings. Since the project was for quality improvement purposes, it did not include audio recording or transcription of interviews, which may have affected the depth and accuracy of the data captured. To mitigate this, the interviewer was trained and experienced in conducting qualitative interviews and capturing field notes, and follow-up clarification from participants was sought as needed. Investigator triangulation was also employed to enhance the rigour of the analysis. Finally, accountability emerged as a key theme during the first round of analysis rather than being a focus of the study from the outset. As a result, the interviewer was limited in their ability to probe participants’ responses directly related to accountability.

## Conclusion

Shared accountability is difficult to operationalize in integrated care, requiring attention not just to formal structures but to how people interpret and navigate accountability in practice. This study identified six interrelated factors that shape these experiences.

OHTs and similar models can strengthen shared accountability by implementing three practical recommendations. First, move beyond broad role descriptions by jointly clarifying key tasks and then creating an RACI matrix—a tool that specifies who is Responsible, Accountable, Consulted, and Informed for each one—to make expectations for backbone and partner leaders more explicit. Light-touch accountability mechanisms, such as progress reports or brief verbal updates in meetings, can make role commitments and contributions more visible without overburdening smaller partners. Second, foster trust and psychological safety by having leaders model vulnerability, for example, through openly acknowledging challenges in their own organizations or asking for peer support to promote shared learning. Third, incorporate structured reflection and dialogue around the guiding questions outlined in Table 1, using them as prompts in meetings to surface tensions, align perspectives, and reinforce shared accountability practices. Together, these steps offer a practical foundation for clarifying expectations, balancing power dynamics, and reinforcing a collective identity, while remaining feasible within the resource constraints of integrated care networks.
